# Financial sustainability of screening, brief intervention, and referral to treatment programs in emergency department settings (part of Economics of SBI symposium)

**DOI:** 10.1186/1940-0640-10-S2-O15

**Published:** 2015-09-24

**Authors:** Alexander J Cowell, William N Dowd

**Affiliations:** 1 RTI International, Research Triangle Park, NC, 19124, USA

## Background

Substance misuse is a leading public health concern in the United States. Emergency departments (EDs) are thought to be an efficient environment in which to deliver screening, brief intervention, and referral to treatment (SBIRT) because a relatively high proportion of patients present with substance misuse. We developed a discrete event simulation model to track SBIRT program costs and revenues in an ED. The results inform the extent to which SBIRT can be sustained by insurance reimbursement in EDs.

## Material and methods

Model parameters are based on nationally-representative public data sources and administrative, observational, and interview data gathered on the Substance Abuse and Mental Health Services Administration (SAMHSA)'s third cohort of SBIRT grantees. SBIRT costs and revenues for approximately 52,000 patients over one year were modeled for a representative ED/trauma center. Results from hypothetical policy scenarios were compared to a base case.

## Results

In the base case and all scenarios, the SBIRT program in an ED setting faced an annual deficit. Under the base case, the program generated $235,420 in revenue and incurred $449,504 in costs, leading to a deficit of $214,084. Populations with higher rates of insurance and/or higher rates of risky substance use led to greater program revenue, as did a higher rate of prescreen completion.

## Conclusions

Under current model assumptions, SBIRT in ED/trauma settings is unlikely to generate enough revenue to cover all associated costs. However, the program may be financially sustainable if the host hospital system were to subsidize some components of program costs. Future work in this area will extend the model to other healthcare settings (e.g., ambulatory clinics) and account for provider heterogeneity.

